# Supplementary material for: Content-rich biological network constructed by mining PubMed abstracts
Source: BMC Bioinformatics. 2004 Oct 8;5:147. doi: 10.1186/1471-2105-5-147 (PMC528731; doi:10.1186/1471-2105-5-147)
Supplement: Additional File 5 — The original Chilibot query results of the term "long-term potentiation (LTP)" and 22 other terms, limiting the latest references analyzed to the years 1990, 1995, 2000, and 2004. [file 1471-2105-5-147-S5.bz2 › chilibotAdditionalFile5/ltp2004/html/AMPA_TAU.html]

 


 **AMPA** and **TAU** 
  
Found 37 abstracts in PubMed,  **30 abstracts were retrieved and analyzed**.  


---

 Search Google  |
 PDF files only 
|  EDU domain only 

---

**Interactive relationship** (e.g. stimulation, inhibition, etc)

- Perfusion with the NMDA receptor antagonist 3 R 2 carboxypiperazin 4 yl propyl 1 phosphonic acid 1 mM or the  **AMPA**  kainate receptor antagonist dinitroquinoxaline dione DNQX 1 mM attenuated the increases produced by PDC 4 mM on DA, GABA, and  **Tau** , and decreases in DOPAC and HVA.  Ref: 9326276 J Neurochem, 1997
- Frequency ratio of NMDA versus  **AMPA**  sEPSCs in slices was correlated with the weighted time constant of decay  **tau**  w of NMDA sEPSCs and decreased with development along the reported decrease of  **tau**  w.  Ref: 11877500 J Neurophysiol, 2002
- Both NMDA and  **AMPA**  kainate receptors are involved in this  **tau**  gene overexpression.  Ref: 9260742 J Neurosci Res, 1997
- Deactivation of  **AMPA**  receptors assessed from motoneuron excitatory postsynaptic currents at 70 mV was independent of postnatal age, with  **tau**  fast =0.88 0.35ms A fast =78.2 11.8% and  **tau**  slow =6.  Ref: 10924957 Neuroscience, 2000

**Parallel relationship** (e.g. studied together, co-existance, homology, etc.)

- An alpha amino 3 hydroxy 5 methylisoxazole 4 propionic acid  **AMPA**  kainate antagonist, dinitroquinoxaline dione 100 microM, blocked SRIF induced DA and  **Tau**  release and reduced that of Asp, Glu, and GABA.  Ref: 9523593 J Neurochem, 1998
- A role for the VTA in the motor responses elicited by dopamine,  **AMPA**  or DAMGO microinjection into the shell, core or VP was evaluated by microinjecting the  **tau**  aminobutyric acidB agonist baclofen into the VTA to inhibit neuronal activity.  Ref: 8627524 J Pharmacol Exp Ther, 1996
